# Swimming Speed of The Breaststroke Kick

**DOI:** 10.2478/v10078-012-0087-4

**Published:** 2012-12-30

**Authors:** Marek Strzała, Piotr Krężałek, Marcin Kaca, Grzegorz Głąb, Andrzej Ostrowski, Arkadiusz Stanula, Aleksander Tyka

**Affiliations:** 1 Department of Theory and Methodology of Water Sports, University School of Physical Education, Cracow, Poland.; 2 Department of Physiotherapy Faculty of Motor Rehabilitation University School of Physical Education, Cracow.; 3 Department of Theory and Methodology of Water Sports, University School of Physical Education, Cracow, Poland.; 4 Department of Physiotherapy Faculty of Motor Rehabilitation University School of Physical Education, Cracow, Poland.; 5 Department of Theory and Methodology of Water Sports, University School of Physical Education, Cracow, Poland.; 6 Department of Sports Theory, The Jerzy Kukuczka Academy of Physical Education, Katowice, Poland.; 7 Department of Physiology and Biochemistry, University School of Physical Education, Cracow, Poland.

**Keywords:** swimming, breaststroke kick, kinematic analysis

## Abstract

The breaststroke kick is responsible for a considerable portion of the forward propulsion in breaststroke swimming. The aim of this study was to measure selected anthropometric variables and functional properties of a swimmer’s body: length of body parts; functional range of motion in the leg joints and anaerobic power of the lower limbs. Chosen kinematic variables useful in the evaluation of swimming performance in the breaststroke kick were evaluated. In the present research, swimming speed using breaststroke kicks depended to the largest extent on anaerobic endurance (0.46, p < 0.05 partial correlations with age control). In addition, knee external rotation and swimming technique index had an impact on swimming speed and kick length (both partial correlations with age control 0.35, p < 0.08). A kinematic analysis of the breaststroke kick hip displacement compatible with horizontal body displacement was significantly negatively correlated with foot slip in the water opposite to body displacement (partial correlations: with leg length control −0.43, p < 0.05; with shank length control −0.45, p < 0.05, respectively). Present research and measurements of selected body properties, physical endurance and kinematic movement analysis may help in making a precise determination of an athlete’s talent for breaststroke swimming.

## Introduction

In breaststroke swimming, according to the International Federation for Swimming -FINA (Fédération Internationale de Natation Amateur), rules that do not apply to any other swimming style determine the stroke cycle from the start and throughout the race; the stroke cycle must consist of an arm stroke and a leg kick that occur in sequence. During each complete cycle, the arm stroke and the leg kick produce similar propulsion forces. In tethered swimming, for example, the peak and mean values of tether forces measured during the propulsion caused by the upper and lower extremities were similar (Yeater et al., 1980).

Early research on advanced kinematic analysis proved that the leg kick is the largest propulsive force of the stroke and that it occurs as the third propulsive force in the breaststroke movement cycle (Mason et al., 1989). The first of the forces, the 'catch', was associated with the hand pull. The second propulsive stage was hypothesised to be the swimmer catching the wave produced by the swimming action itself with the relatively large surface of the trunk moving forwards against a still, incompressible water volume (Mason et al., 1989). It was also recognised in a study by Kippenhan (1991) that the breaststroke kick, especially the whip kick, makes a major contribution to the forward propulsion in this style.

Moreover, Leblanc et al. (2007) observed that in elite swimmers, a higher peak forward velocity was reached during arm propulsive phases when swimming distances of 50 and 100 m at race-pace. Maglischo (2003) explained that swimmers accelerated their bodies for a longer time with their arms than they did with their legs, but the kick is clearly a dominant propulsive force in the breaststroke. Propulsion from the kick begins when the forward velocity is the lowest in the stroke cycle, whereas the legs begin to accelerate the body forwards together with the arms when they are already traveling much faster. This occurs at the highest breaststroke velocities.

A correctly performed whip kick is challenging to execute. Difficulties are generally associated with complicated hip, knee, and ankle joint motion sequences (Kippenhan, 1991). A high range of motion, flexibility and above-average musculoskeletal forces during the movement produce powerful whip kick propulsion. The structure of one’s legs and feet may also play a role in becoming a good breaststroker, described by others as “walking like a duck” (Bixler, 2005). These characteristics are visible to the coach and may be measured. Indeed, in a study by Kippenhan (2002), it was noted that in a group of collegiate varsity and recreational swimmers, the more skilled ones had a larger external knee rotation. In an investigation conducted by Jagomägi and Jürimäe (2005) on a large sample of 125 female swimmers, the swimming velocity in a 100 m breaststroke performed using a kickboard and legs was significantly influenced by the flexibility of the hip, knee and ankle. This swimming velocity was also significantly related to the body height and body mass and to the standing broad jump. Gerard et al. (1986) showed a statistically significant direct dependence between anaerobic power measured by vertical jump results and type II fibre content in the *vastus lateralis* muscle with a higher efficiency in short-rather than long-distance swimming.

Considering these data, we measured several anthropometric indices and functional properties of the breaststroke: length of body parts, range of motion in the leg joints and anaerobic power of the lower limbs. We also constructed kinematic indices useful in the evaluation of swimming performance of the breaststroke kick. We hypothesised that the above-mentioned indices may influence the basic swimming technique variables: stroke rate and stroke length of the breaststroke kick and swimming speed using a kickboard and legs only.

## Methods

### Participants

Volunteers were recruited from two sport schools and from a university swimming club. They or their parents signed a written informed consent before taking part in the research, which was approved by the Bioethics Committee in Cracow. The 27 swimmers representing regional and national sports levels trained twice a day, six times a week. The swimmers were 15.7 ± 1.98 years old; 26 were between 14 to 19 years old, and one was 21 years old. The average body height was 180.1 ± 7.21 cm, and average body mass was 70.3 ± 9.35 kg. The subjects specialised in the breaststroke and in the individual medley.

### Anthropometry

The anthropometric data that was gathered made it possible to determine the swimmers’ somatotypes according to Carter–Heath (1990). Within the sample, 18 swimmers represented the mesomorphic somatotype, and 9, the ectomorphic one. The measurements were conducted with appropriate anthropometric apparatus (Sieber Hegner Maschinen AG, Switzerland; the Harpenden-type skinfold callipers with a constant pressure of 10 g/cm^−2^ were used). The percentage of body fat was 10.21 ± 2.41%. Lower extremity and shank lengths (Lg-L and Sh-L, respectively) were measured in a recumbent position from the trochanter major and head of the fibula, respectively, to the toes with full plantar flexion.

### Leg joint mobility

The range of motion for each joint in the lower extremity was measured, preceded by a warm-up similar to that used in competition conditions without specific stretching, using a plastic goniometer by an experienced physiotherapist. A reference angle of 0º was assumed for the sagittal plane orientation. Hip joint internal rotation (Hip-Int) was measured in the sitting position by bending to a maximal bending angle close to 90º. Knee external rotation (Knee-Ext) was measured in a horizontal position lying prone by bending the joint to an angle close to 90º . Ankle supination range (Ankle-Ext) was also measured in the same position, but with mechanical knee and shank immobilisation. The measurement reliability was high; the range was 1º for repeated measurements. The mean value of the range of motion for the right and left hip joints was registered. The same procedure was conducted for knees and ankles.

### Anaerobic endurance

Maximal power and work output during vertical jumps performed in a 15-second period (15s-VJ) were recorded using the Opto Jump (Italy) apparatus. The height of the centre of gravity during the vertical jump (CMJ) was assessed using the jump duration formula 1. Work during the jump (W) was calculated according to formula 2.

(1) $$\Delta h=\frac{gt_{l}^{2}}{8}$$

(2) $$W=\mathrm{mg}\Delta h=\frac{mg^{2}t_{l}^{2}}{8}$$

where:

*WL*: work during the jump [J]
*g*: gravitational acceleration [*m s*^−2^]
*Δh*: elevation of the centre of gravity [*m*]
*m*: body mass [kg]
*t_l_*: jump duration [s]

The 50 m all-out swimming speed tests were carried out in a 25 meter-long swimming pool, with the start in the water using a kickboard and breaststroke kick only.

All swimmers used the same standard kickboard, which was positioned against the upper part of the body identically for every swimmer. The so-called high grip of the kickboard was used.

### Underwater recordings

The swimmers’ body movements were recorded using a Canon Legria HV40 (Japan) camcorder and an underwater camera, a Sony Color Submergible Camera IP:68 (Japan). Video recordings were made with the frequency of 50 frames per second. The recording took place from the side with the camcorder installed on a portable trolley moving along the swimming pool border parallel to the swimmer. A person trained to operate the above-mentioned device pulled the trolley in both directions along the swimming pool in such a way as to not to cross the perpendicular lines between the swimmer’s head and the lens at the front and the toes of the stretched legs at the back. The underwater camera was mounted to the lower arm of the trolley and emerged in water approximately 1 meter below the surface at a distance of approximately 5 meters from the swimmer’s lane.

### Basic video analysis of breaststroke kick swimming

The video analysis made it possible to determine the moments in which characteristic events of the swimming cycle occurred, i.e., the start of propulsive movement of the lower extremities at the beginning of the backward leg movement at the moment the stretching began in the knee joint.

The video analysis made it possible to identify the characteristic cyclic elements of leg movements in the breaststroke kick: kick rate (KR) and kick length (KL), which was calculated as the ratio between KR and the average swimming speed (V) for the 50 m distance.

The duration of the race Δ*t_i_* and times within separate sectors were measured with a stopwatch with accuracy of 0.01 s.

The following parameters were used to assess swimming technique during each analysed 25 m swim (i=1, 2):

1. Swimming speed: V_i_=25 m / Δt_i_ [m·s^−1^],
2. Kick rate (KR_i_), calculated as the reciprocal of the arithmetical average of the duration of three analysed swimming cycles: KR_i_=1/T_i_ [ckl·min^−1^],
3. Kick length (KL_i_), calculated as the average speed over KR_i_ ratio: KL_i_=V_i_/KR_i_ [m].

The results of swimming technique variables KR and KL and the kinematic indices described below are presented as the means calculated from six full leg movement cycles, from which 3 were identified in the 5 – 20 m sector and the other 3 were identified in the 30 – 40 m sector of the 50 m distance.

### Kinematic analysis of the breaststroke kick

The analysis of the ankle and hip movement was performed using KA Video software - R. Schleihauf, San Francisco State University (2006). At first, a scaling coefficient for every athlete was obtained using anthropometric measurements. Then, the positions of markers located on ropes on both sides of the lane and the positions of the hip and ankle joints were identified in every recorded frame. A global reference system was selected with the origin at the midpoint between lane rope markers. Afterwards, global coordinates of the hip and ankle joints were calculated for every film frame. The phase-ending event, when the distance between the hip and ankle is maximal, was identified for every movement cycle. The relative displacements of the ankle and hip joints d_A–H_ [m] and the displacements of these joints in the global reference system (d_A_ [m] - feet slip, d_H_ [m] - body forward movement) during the whole phase were calculated. Finally, quotients were calculated for the ankle and hip displacements and their relative displacements d_rA_ =d_A_/d_A–H_ [%] and d_rH_=d_H_/d_A–H_ [%].

### Statistical methods

Means and standard deviations were calculated. To determine the relationship between swimming speed with somatic, functional, swimming technique variables and anaerobic capacity indices, the partial correlations, controlling for age, were computed. The relationships between kinematic indices (Hip displacement and Ankle displacement) were also partial correlations computed by controlling for leg (Lg-L) and shank length (Sh-L), knee rotation and anaerobic capacity indices. The Pearson correlation coefficient was computed between KL and lower extremity indices Lg-L and Sh-L. A normal distribution of data was registered. All tests were conducted with STATISTICA 6.1 software (StatSoft, Inc). The significance level was set at *p* < 0.05.

## Results

Mean swimming speed using the breaststroke kicks for a distance of 50 m (V50) was 1.10±0.06 m·s^−1^. The partial correlation measurements with age control between somatic and functional variables are shown in Table 1. The anaerobic endurance index showed an average relation with swimming speed.

Partial correlations on the verge of statistical significance between the KL index and the knee joint movement range were noted (Table 2). The KL correlation, without age normalisation, with lower extremity length indices Lg-L and Sh-L showed a dependence on the verge of statistical significance of 0.34, p=0.08, and 0.37, p<0.06, respectively.

A dependence on the verge of statistical significance was reached between the knee joint external rotation range and swimming speed with age control (Table 2).

Kinematic indices of the propulsion phase of breaststroke kicks with age control in most cases significantly influenced V50 values and SL values (Table 3).

The KL index of the breaststroke kick technique analysis did not show other significant correlations.

Significantly greater hip displacement in the propulsive movement was negatively correlated with foot slip in the water with lower extremity length indices control, Lg-L and Sh-L (Table 4).

## Discussion

The kinematic analysis of breaststroke kicks in our study may support observations of swimming specialists regarding the fact that a swimmer’s talent is recognised by the so-called feeling of the water, which is manifested by precise and strong propulsive movements without excessive extremities slip-sink through the water. In our measurements, hip displacement (d_H_) compatible with horizontal body displacement was not positively related to ankle joint displacement (d_A_) opposite to body displacement. Moreover, it was stated that these dependencies, d_H_ to foot slip (d_A_) with control of lower extremity length indices Lg-L and Sh-L, are negatively correlated.

Vorontsov and Rumyantsev (2000) in their literature review stated that leg actions are able to create greater hydrodynamic forces than arm actions for the following reasons: legs possess significantly greater propelling surface area, and the muscle groups of the legs are significantly stronger than those of the arms. However, in swimming, these features play a minor role in creating effective propulsive forces (Vorontsov and Rumyantsev, 2000). The results of our research support these statements.

In the present study, swimming speed using breaststroke kicks depended primarily on the anaerobic endurance index (15s-VJ). In addition to the 15s-VJ, the knee external rotation (Knee-Ext) and the swimming technique index KL had a direct impact on swimming speed.

To generate an above-average propulsive force in multidimensional breaststroke kicks, besides muscular power Falk et al. (2004), Platanou (2005), it is vital to have a wide range of movement in each of the lower extremity joints. In the research of Jagomägi and Jürimäe (2005), external knee rotation had more of an influence on swimming speed in a 100 m breaststroke swim using a kickboard than did external hip rotation or ankle supination. All these dependencies on the average level were statistically significant in the group of female swimmers. Jagomägi and Jürimäe (2005) stated that the more flexible the ankles and knees are, the easier it is to keep the kick narrow and kick back rather than out. In contrast, the entire hip and ankle joint range of movement available in the breaststroke was not used by the most skilled swimmers in the Kippenhan’s (2002) observations. However, they noted that the more skilled the subjects were, the greater the external knee rotation during the breaststroke kick, external knee rotation towards the end of the downsweep in particular. According to them, increasing the external knee rotation during this phase was essential for maintaining a more propulsive foot orientation for as long as possible.

In research on breaststroke kicks conducted by Matheson et al. (2011) in female NCAA Division I swimmers, four different approaches were observed, based on variations of movement of lower extremity segments. The authors present examples: two participants predominantly used a thigh and foot approach. Three participants used mainly adduction with most of the contribution coming from the thigh shank. Three others performed the kick from the knee down, using mainly shank and foot velocities, while four participants displayed a more distributed sequence by using significant contributions to velocity from all three segments.

## Conclusions

The present research and measurements of selected body properties, physical endurance testing and kinematic movement analysis may help in precise detection of an athlete’s talent for the breaststroke. The observed dependencies of anaerobic endurance (15s-VJ), kick length, as well as the knee joint movement range in the lateral plane on speed with regard to the breaststroke kicks form an important conclusion which may have an impact on swimming technique shaping as well as swimmers body abilities implicating speed and movement trajectory of the legs in breaststroke. Furthermore, our measurements of hip displacement (d_H_) compatible with horizontal body displacement which was not positively related to foot slip (d_A_) may be considered helpful in the assessment of breaststroke kick efficiency.

## Figures and Tables

**Table 1:** Somatic properties: Lg-L, Sh-L, anaerobic endurance index (15s-VJ), kick length (KL) and kick rate (KR) of the movement cycle of lower extremities and their partial correlations with swimming speed (V50) with age control. Statistically significant (p<0.05) correlations are marked with an asterisk. Correlations on the edge of significance (p<0.08) are marked in italics.

Indices	Lg-L [cm]	Sh-L [cm]	15s-VJ [J]	KL [m]	KR [cycle·min^−1^]

	105.5±4.20	61.8±3.03	3787.1±574.66	1.26±0.15	53.2±5.69
					
V 50 [m·s^−1^]	0.22	0.09	0.46 ^*^	*0.35*	0.05

**Table 2:** Hip, knee and ankle joint movement range in the lateral plane and their partial correlations with swimming speed (V50) with age control. Correlations on the edge of significance (p<0.08) are marked in italics.

Indices	Hip-Int [º]	Knee-Ext [º]	Ankle-Ext [º]

	35.3±7.25	44.0±8.48	30.6±7.06
			
V 50 [m·s^−1^]	0.08	*0.35*	0.09

**Table 3:** Kinematic indices of the breaststroke kick propulsion phase and their partial correlations with swimming speed (V50) and leg movement cycle length (KL) with age control. Statistically significant correlations are marked as follows: p<0.05^*^, p<0.01^**^.

Indices	d_H_ [m]	d_A_ [m]	d_rH_ [%]	d_rA_ [%]	VH_max_ [m·s^−1^]	VH_aver_ [m·s^−1^]

	0.32±0.03	0.29±0.04	0.52±0.05	0.48±0.05	1.54±0.11	1.16±0.08
						
V 50 [m·s^−1^]	0.67 ^**^	−0.19	0.46 ^*^	−0.46 ^*^	0.58 ^**^	0.78 ^**^
KL [m]	0.63 ^**^	−0.23	0.46 ^*^	−0.46 ^*^	0.21	0.39 ^*^

**Table 4:** Partial correlations between kinematic indices: Hip displacement (d_H_) and ankle displacement (d_A_) with control of somatic indices L-gL, Sh-L, anaerobic endurance index (15s-VJ) and range of knee joint external rotation (Knee-Ext). Statistically significant correlations p< 0.05 are marked with an asterisk.

Indices	d_A_ [m]	d_A_ [m]	d_A_ [m]	d_A_ [m]

	controlling Lg-L [cm]	controlling Sh-L [cm]	controlling Knee-Ext [º]	controlling 15s-VJ [J]
				
d_H_ [m]	−0.43 ^*^	−0.45 ^*^	−0.20	−0.19
